# Surgical resection of a primary cardiac angiosarcoma and reconstruction of involved right atrium

**DOI:** 10.1093/jscr/rjaf1046

**Published:** 2026-01-08

**Authors:** Jiahong Xie, Peiwen Mai, Yang Wu, Xiaowu Wang

**Affiliations:** Department of Cardiovascular Surgery, Zhujiang Hospital, Southern Medical University, No. 253 Industrial Avenue, Haizhu District, Guangzhou, Guangdong Province 510280, China; Guangzhou Panyu District Blood Center, No. 25 Huizhong Street, Panyu District, Guangzhou, Guangdong Province 511442, China; Department of Cardiovascular Surgery, Zhujiang Hospital, Southern Medical University, No. 253 Industrial Avenue, Haizhu District, Guangzhou, Guangdong Province 510280, China; Department of Cardiovascular Surgery, Zhujiang Hospital, Southern Medical University, No. 253 Industrial Avenue, Haizhu District, Guangzhou, Guangdong Province 510280, China

**Keywords:** primary cardiac angiosarcoma, surgical resection, multimodality imaging, immunotherapy

## Abstract

Primary cardiac angiosarcoma is an extremely rare malignant neoplasm, typically showing terrible prognosis, the gold standard treatment is surgical resection. We present a case of a 41-year-old woman who was diagnosed with a large right atrium, presenting with complaints of chest pain and shortness of breath for 2 weeks. Multimodality imaging results suggest a cardiac angiosarcoma. The patient underwent successful radical resection and single-stage reconstruction of the right atrium and superior vena cava under normothermic cardiopulmonary bypass with a beating heart. Histopathology confirmed the diagnosis of primary cardiac angiosarcoma. Based on the genetic results, tiragolumab was used for postoperative immunotherapy. Fortunately, no evidence of recurrence of angiosarcoma was showed 4 year after surgery. Radical surgical resection with reconstruction of the resected heart structures was the only possible salvage option for giant angiosarcoma, which led to hemodynamic instability. Followed by chemotherapy, this radical approach may prolong survival.

## Introduction

Primary cardiac angiosarcoma is an exceptionally rare and notoriously aggressive malignancy [1], representing the most common histological subtype of primary malignant cardiac tumors. The tumor demonstrates a predilection for the right atrium and ventricle [2], characterized by rapid, invasive growth and a high propensity for metastasis, particularly to the lungs, often at the time of diagnosis. Clinical presentation is often insidious, with non-specific constitutional symptoms such as dyspnea, chest pain, and fatigue leading to frequent delays in diagnosis [3]. While advances in multimodality imaging have improved detection, establishing a timely diagnosis remains challenging [4].

Surgical resection remains the cornerstone of treatment and the only potential for cure. However, complete resection is complex due to the tumor’s frequent invasion of vital cardiac structures, necessitating sophisticated reconstruction. This report details a case of a large right atrial primary cardiac angiosarcoma successfully managed with radical surgical resection and reconstruction, highlighting this approach as a critical salvage option in selected patients.

## Case presentation

**Figure 1:**
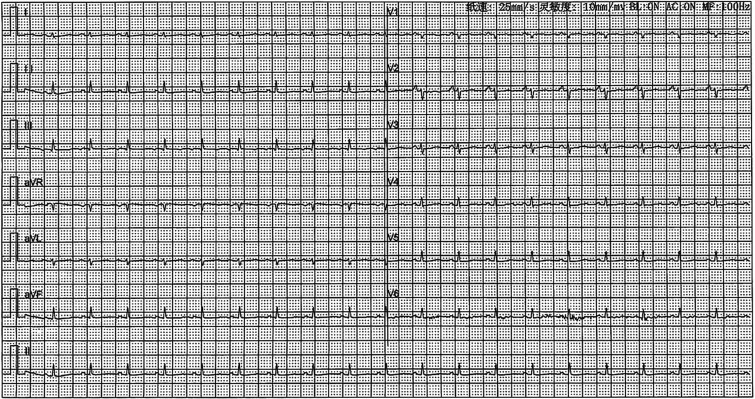
Electrocardiography demonstrated sinus tachycardia and low-voltage complexes.

**Figure 2:**
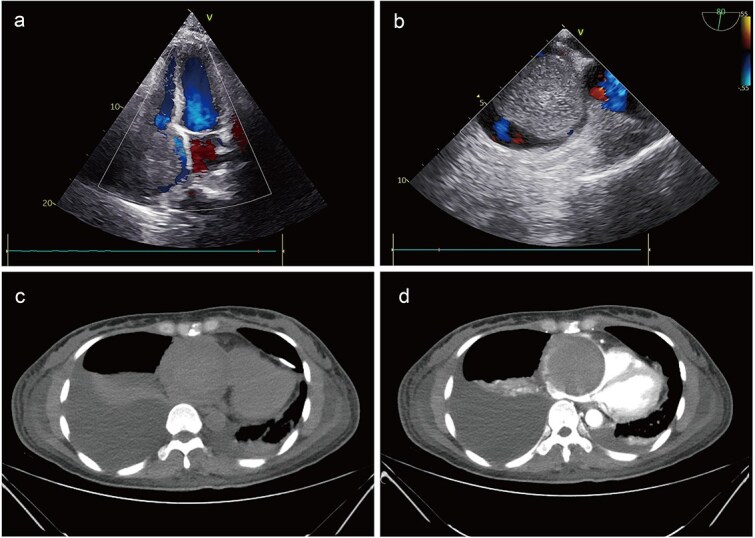
Preoperative imaging. Echocardiography identified a large mass, nearly occupying the entire right atrial cavity (a) and (b). Contrast-enhanced computed tomography showed a right pleural effusion and a right atrial occupying lesion (c) and (d).

**Figure 3:**
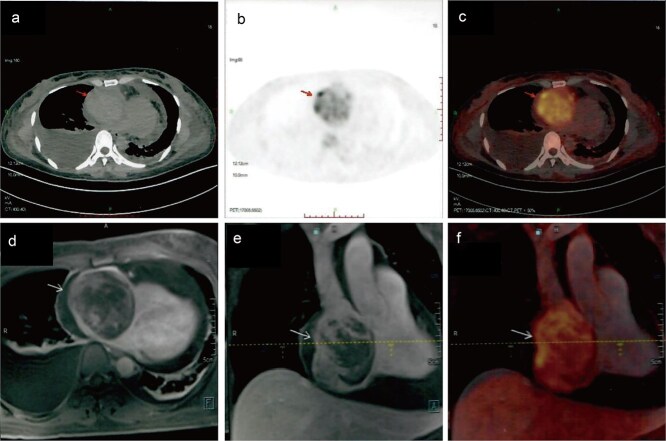
Metabolic imaging. PET-CT (a) and (b) and PET-MR (c) and (d) demonstrated a hypermetabolic soft tissue mass within the right atrium, highly suggestive of primary cardiac angiosarcoma.

A 41-year-old female was admitted to our hospital with chest tightness and dyspnea for the past 2 weeks. Physical examination revealed bilateral lower limb edema without cardiac murmurs or jugular venous distention. Laboratory findings were notable for hypoalbuminemia (30 g/L) and an elevated D-dimer level (6.99 mg/mL), without remarkable blood routine examination and serum chemistry. Electrocardiography demonstrated sinus tachycardia and low-voltage complexes (Fig. 1). Echocardiography identified a large, well-demarcated hypoechoic mass (82 × 54 × 58 mm), nearly occupying the entire right atrial cavity (Fig. 2a and b). Contrast-enhanced computed tomography confirmed right pleural effusion and a right atrial occupying lesion (Fig. c and d). Subsequent right thoracentesis revealed exudative fluid with no evidence of malignancy on cytological examination. Further metabolic imaging with PET-CT (Fig. 3a and b) and PET-MR (Fig. 3c and d) demonstrated a hypermetabolic soft tissue mass within the right atrium, highly suggestive of primary cardiac angiosarcoma, with no signs of distant metastasis.

**Figure 4:**
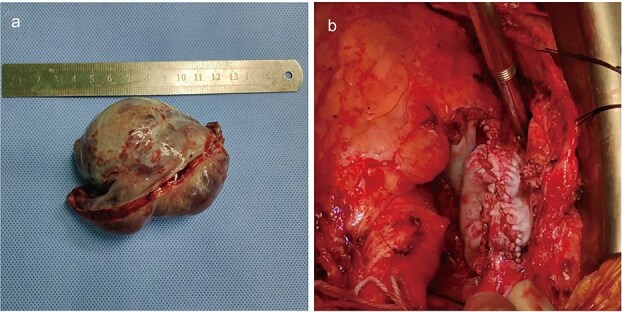
Intraoperative imaging. The mass was completely resected (a). The involved portions of the right atrial wall and venae cavae, was performed, followed by reconstruction using bovine pericardium (b).

In light of the high suspicion for malignancy, considerable tumor size, and significant risks of thromboembolism and tricuspid valve obstruction, a multidisciplinary team decision was made to proceed with surgical resection. Under normothermic cardiopulmonary bypass with a beating heart, intraoperative exploration confirmed tumor origin from the right atrial free wall with extension into both the superior and inferior venae cavae. Given the extensive involvement, a radical en bloc resection of the tumor (Fig. 4a), encompassing nearly the entire right atrial free wall and the involved segments of the venae cavae, was performed. This was followed by an anatomical reconstruction of the right atrium and superior vena cava using bovine pericardium (Fig. 4b).

**Figure 5:**
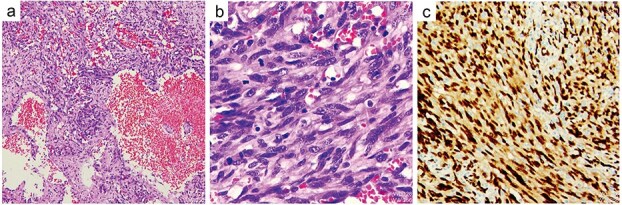
Histopathological examination. The resected specimen exhibiting marked cellular atypia, frequent mitotic activity, and prominent vasoformative features (a) and (b). Immunohistochemical staining was positive for ERG (c).

Histopathological examination of the resected specimen confirmed a malignant neoplasm exhibiting marked cellular atypia, frequent mitotic activity, and prominent vasoformative features (Fig. 5a and b). Immunohistochemical staining was positive for Vimentin, CD34, CD31, ERG (Fig. 5c), FLI-1, and INI-1, with focal SMA expression, while negative for MyoD1, Myogenin, Desmin, cytokeratin, CK5/6, calretinin, and S100. The Ki-67 proliferation index was 40%, supporting the definitive diagnosis of primary cardiac angiosarcoma.

**Figure 6:**
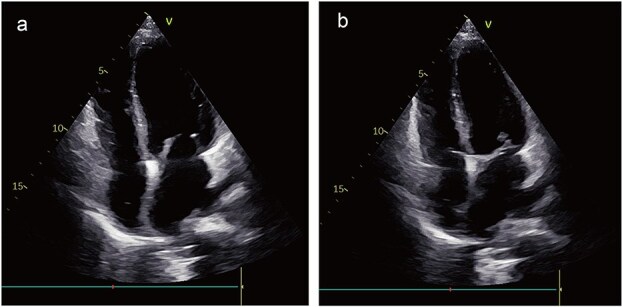
Postoperative images. The echocardiogram indicated no signs of local recurrence (a) and (b).

During surveillance at 3 years postoperatively, echocardiography showed no local recurrence; however, chest CT identified multiple pulmonary nodules suggestive of metastatic disease. Genetic profiling demonstrated PD-L1 positivity, prompting initiation of immunotherapy with tislelizumab. At the 4-year follow-up, the patient remained clinically stable with no echocardiographic evidence of local recurrence (Fig. 6a and b) and continues under close monitoring.

## Discussion

Primary cardiac angiosarcoma, while accounting for only 0.001%–0.3% of all cardiac tumors, represents the most frequent primary cardiac malignancy, comprising 30% of malignant cases [1, 6]. The right atrium is the most common site of origin, often with local invasion into surrounding structures like the pericardium, venae cavae, and tricuspid apparatus. Clinical presentation is non-specific, frequently leading to delayed diagnosis. The prognosis remains poor, with a mean survival of merely 3.8 ± 2.5 months without intervention [7], emphasizing the critical need for aggressive management.

Multimodality imaging is indispensable for diagnosis and preoperative planning [4]. Echocardiography serves as the initial screening modality for tumor detection, providing assessment of location, dimensions, and functional consequences. Contrast-enhanced computed tomography typically reveals a heterogeneously enhancing mass with infiltration. Cardiac magnetic resonance imaging offers superior soft-tissue contrast that help distinguish tumor from thrombus and delineate the extent of myocardial and pericardial invasion. PET-CT or PET-MR further contributes through metabolic activity assessment and comprehensive screening for distant metastases. The synergistic application of these imaging techniques facilitates earlier and more accurate diagnosis, thereby promoting timely implementation of appropriate therapeutic interventions.

Surgical resection constitutes the cornerstone of treatment and represents the most significant prognostic determinant for survival. The primary objectives encompass complete resection with curative intent and alleviation of life-threatening obstructions causing hemodynamic compromise [8]. As demonstrated in our case, management typically necessitates en bloc resection of the right atrial wall along with involved structures, followed by meticulous reconstruction using materials such as bovine pericardium to restore cardiac geometry and function. The technical novelty of our case lies in the performance of this extensive resection and complex reconstruction under normothermic cardiopulmonary bypass with a beating heart. This approach offers the potential advantages of avoiding myocardial ischemic injury and facilitating real-time assessment of the reconstructed cardiac geometry and function. Although achievement of histologically complete margins proves challenging and is reported in only ~25% of cases, aggressive resection remains justified even with microscopic residual disease to ameliorate symptoms and improve survival [9]. While the optimal outcome associated with complete surgical resection, the substantial risk of both local recurrence and distant metastasis necessitates consideration of, targeted medicines and immunotherapy [10]. Ultimately, a comprehensive multimodal approach, carefully tailored to individual disease extent and functional status, offers the optimal opportunity for prolonged survival.

## Conclusion

Given the poor prognosis of primary cardiac angiosarcoma, prompt surgical resection followed by close surveillance should be undertaken once the diagnosis is established. Multimodality imaging plays a crucial role in both diagnosing primary cardiac angiosarcoma and guiding surgical strategy. Additionally, aggressive immunotherapeutic approaches represent an important modality for improving survival outcomes in patients with primary cardiac angiosarcoma.
